# Recent Advances of Artificial Intelligence Applications in Interstitial Lung Diseases

**DOI:** 10.3390/diagnostics13132303

**Published:** 2023-07-06

**Authors:** Konstantinos P. Exarchos, Georgia Gkrepi, Konstantinos Kostikas, Athena Gogali

**Affiliations:** Respiratory Medicine Department, University of Ioannina School of Medicine, 45110 Ioannina, Greece; kexarcho@gmail.com (K.P.E.); geogkrepi@gmail.com (G.G.); ktkostikas@gmail.com (K.K.)

**Keywords:** interstitial lung diseases, diffuse parenchymal lung diseases, artificial intelligence, machine learning, deep learning

## Abstract

Interstitial lung diseases (ILDs) comprise a rather heterogeneous group of diseases varying in pathophysiology, presentation, epidemiology, diagnosis, treatment and prognosis. Even though they have been recognized for several years, there are still areas of research debate. In the majority of ILDs, imaging modalities and especially high-resolution Computed Tomography (CT) scans have been the cornerstone in patient diagnostic approach and follow-up. The intricate nature of ILDs and the accompanying data have led to an increasing adoption of artificial intelligence (AI) techniques, primarily on imaging data but also in genetic data, spirometry and lung diffusion, among others. In this literature review, we describe the most prominent applications of AI in ILDs presented approximately within the last five years. We roughly stratify these studies in three categories, namely: (i) screening, (ii) diagnosis and classification, (iii) prognosis.

## 1. Introduction

Interstitial lung diseases (ILDs), also known as diffuse parenchymal lung diseases, is an umbrella term encompassing more than 300 conditions. The term interstitial refers to the tissue surrounding the air sacs (alveoli) in the lung, bounded by the capillary endothelium and the alveolar epithelium. Most ILDs are often characterized by inflammation and/or fibrosis causing progressive decline in lung function and impaired gas exchange; consequently, patients complain of shortness of breath, cough, fatigue and weight loss. The most common causes of ILDs include exposure to environmental toxins, viral infections and autoimmune conditions, while many are idiopathic.

The diagnosis of ILDs is often challenging due to the diversity of symptoms and the fact that they can mimic symptoms of other respiratory disorders. A thorough medical evaluation, including pulmonary function tests, consecutive imaging studies, bronchoscopy and even biopsy, is often necessary to make a definitive diagnosis. Even after successful diagnosis, most of these exams are regularly collected and reviewed by the treating physician. As for epidemiology, ILDs range from ultra rare to relatively common. Overall, they collectively affect a considerable number of patients, posing a significant disease burden.

**Figure 1:**
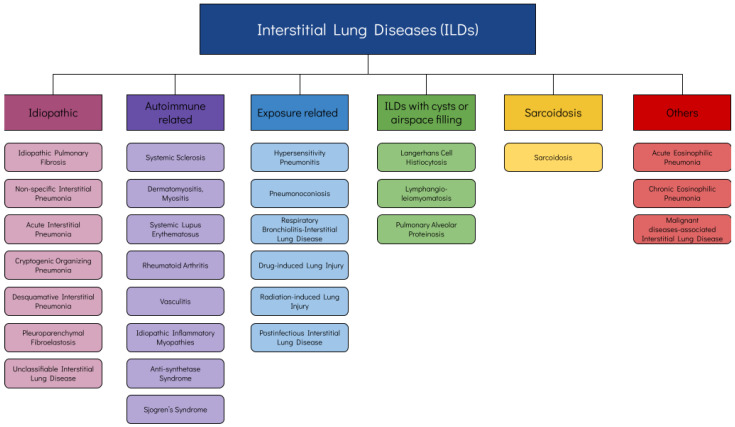
Classification of ILDs.

In terms of classification, there is no unanimous nomenclature; some categorizations distinguish ILDs of known cause from those of unknown etiology. Another common classification identifies the following categories: idiopathic, autoimmune-related, exposure-related (including iatrogenic), interstitial lung diseases with cysts or airspace filling, sarcoidosis and orphan diseases [1]; this classification is shown in Figure 1. Due to their scarcity and heterogeneity, it is difficult to gain expertise; therefore, in several clinical settings, diagnosis remains elusive for a considerable amount of time. Specifically, a recent study has reported that the median time from symptom onset to diagnosis is 7 months, often accompanied by at least one misdiagnosis [2]. Moreover, some of these conditions, due to their etiology, pathophysiology or presentation, may require close collaboration with other medical specialties, e.g., rheumatologists. Even after successful diagnosis, choosing the best treatment for each patient is also debatable, as well as assessing and quantifying progression or response to treatment.

**Figure 2:**
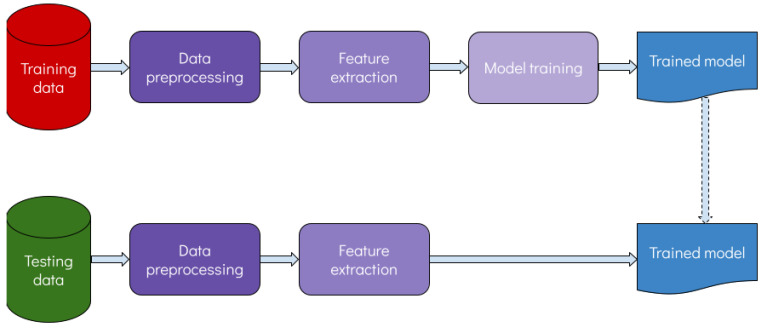
Flowchart of the learning process.

Artificial intelligence (AI) is a relatively old term which has been attracting significant attention during the last few years. AI refers to the technologies that enable machines to perform advanced human-like functions, such as learning, analyzing, seeing, etc., and gain experience as more data become available. AI is a broad field encompassing many different disciplines such as computer science, statistics, software engineering and many others. Machine learning (ML) is a subfield of AI where statistical models are trained to “learn” patterns from complex data in order to perform a specific task. Figure 2 shows a flowchart of the learning process.

**Figure 3:**
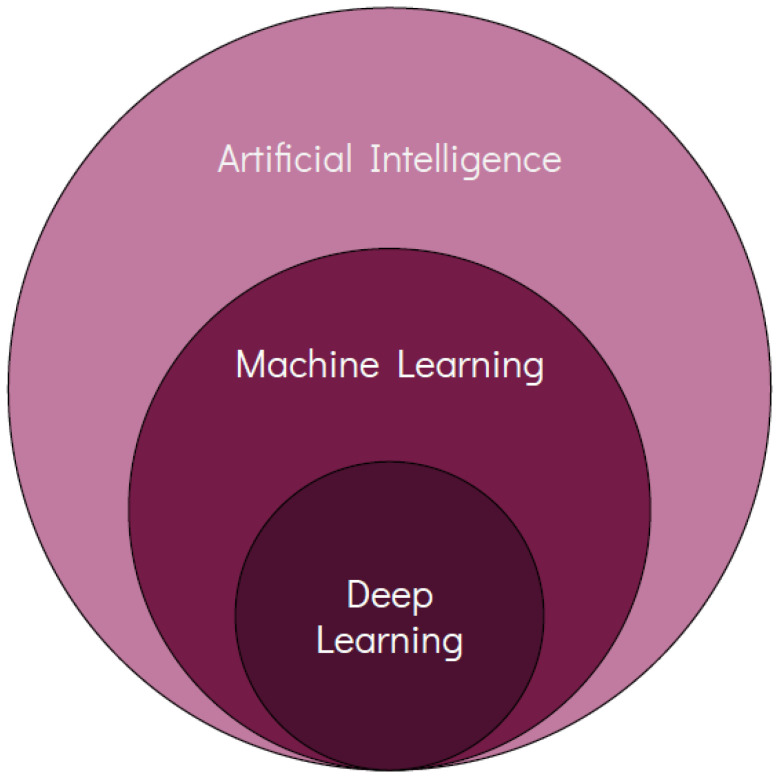
Hierarchy of artificial intelligence, machine learning and deep learning algorithms.

Moreover, another branch of AI that is currently in the spotlight is called deep learning (DL); DL features a complex multi-layer architecture resembling the distributed approach to problem solving that is carried out by the neurons of the human brain (Figure 3). Thanks to its implementation, DL can analyze huge amounts of complex data and has produced some noteworthy applications across several industries, e.g., self-driving cars, language translation, virtual assistants, etc.

Advances in processing power and its wide availability in everyday devices such as home computers and smartphones have contributed significantly towards this direction. In addition, during the last few years, numerous machine learning and even deep learning software tools have been developed that require minimal or no coding skills to perform a certain task. All these advances go hand in hand with the production and storage of huge amounts of complex data from nearly every field of science and elsewhere. The most significant factor in the advent of ML and DL algorithms is the achievement of superior performance compared with traditional methods or algorithms. The ability to interpret or explain the output of some ML algorithms that have been traditionally considered “black-boxes” has certainly contributed towards wider acceptance of such algorithms.

**Figure 4:**
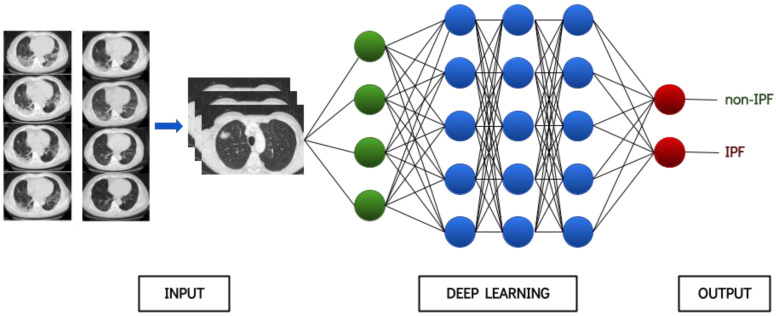
Provisional architecture of a deep neural network.

This issue is specifically true for the utilization of ML algorithms in healthcare applications. Until recently, the lack of reasoning in medical decisions from ML algorithms has led to considerable skepticism that is being gradually alleviated. Moreover, it has become almost unanimously apparent that AI does not substitute medical personnel, but rather works in a decision-support manner, thus facilitating trivial tasks so that doctors can focus more freely and effectively on the patient. As a result, some impressive examples have been reported in the healthcare industry as well, especially with regard to computer vision tasks, at which DL algorithms are profoundly adept. Such examples include skin lesions [3], endoscopic images [4], histopathologic images [5] and radiology images. The latter type of data, especially CT scans, constitutes an integral part in the pursuit of ILDs. DL has revolutionized the way medical images are analyzed and interpreted and has been used in a wide range of medical imaging applications, including segmentation, registration, classification, object detection as well as many others. An exemplary architecture of a deep neural network is shown in Figure 4.

Besides imaging data, ILDs produce other sources of biomarkers that need to be analyzed in conjunction with the findings from the imaging modalities that are integral in the pursuit of ILDs. Recently, there is active interest in biomarkers coming from genomic, proteomic and transcriptomic investigations. Further considering the evolution and variability of the aforementioned data over time, we provide some perspective regarding the complexity of data pertaining to ILDs.

It is evident that ILDs are an ideal candidate for encompassing AI algorithms. In the current literature review, we describe the most prominent and recent applications of AI algorithms in ILD research. The majority is focused on the analysis of imaging modalities; nevertheless, other biomarkers, e.g., volatile organic compounds, gene expression, etc., are also being exploited in all aspects of ILD research: from screening to diagnosis and overall prognosis as well as treatment, aiming for more personalized and effective strategies [6].

## 2. Artificial Intelligence in ILDs

### 2.1. Overview and Rationale

ILDs constitute a complex and heterogeneous set of entities; each entity is often accompanied with large amounts of multiscale and multiparametric data (chest X-rays, CT scans, spirometries, oscillometries, etc.) varying from entity to entity, across patients of the same entity as well as over time. This inherent complexity, coupled with the rarity of some of these diseases, calls for advanced computational models, such as the ones from the field of AI. In the sections that follow, we first present some notable data repositories pertaining to ILDs, and then we discuss a selection of the most recent, innovative and remarkable applications of AI in ILD research.

### 2.2. Data Repositories

A critical issue for the exploration of ILDs, especially with AI algorithms, is the production of high-quality data that are well characterized and annotated. Same as in all healthcare-related repositories, anonymization and data privacy issues are of utmost importance and should be considered thoroughly, even in small-scale private datasets. Below, we briefly mention some data repositories that can and have been used in ILD research. Each dataset contains different types of data for variable ILD conditions and has been deployed by several authors and research groups with varying permutations depending on the scope of the study.

- OSIC data repository: launched by the Open Source Imaging Consortium, the OSIC repository contains approximately 1500 anonymized high-resolution CT scans alongside with clinical data for a wide range of interstitial lung diseases, primarily IPF [7]. The repository contains treatment information, as well as follow-up data and mortality. It was built by a collaboration of experts in the fields of pulmonology, radiology and AI, aiming primarily to facilitate the role of the latter in patient care and precision medicine. A subset of the OSIC data repository containing information regarding pulmonary fibrosis progression has become available from Kaggle, for researchers to develop tools and algorithms aiming to predict lung function decline. The subset repository contains 200 cases with approximately 1–2 years of follow-up.
- ILD Database from medGIFT [8]: a publicly available multimedia collection of ILD cases built from the University Hospitals of Geneva. The database contains high-resolution CT scans with annotated regions of pathological lung areas coupled with clinical parameters from patients with pathologically proven diagnoses of ILDs. Overall, the library contains data from 128 patients affected with 1 of the 13 ILD diseases.
- ILDgenDB [9]: an integrated genetic knowledge resource for interstitial lung diseases, which, as the name implies, contains genetic data about several ILDs. This resource contains literature-curated disease candidate genes enriched with regulatory elements, as well as single nucleotide polymorphisms (SNPs) that have been associated with specific ILDs. For this propose, ILDgenDB is enriched with information from multiple popular genetic resources such as GAD (Genetic Association Database), OMIM (Online Mendelian Inheritance in Man) and GeneCards. The objective of this resource is to pinpoint potential genetic targets related to the pathogenesis, diagnosis, monitoring and treatment of ILDs.
- ILDGDB [10]: a similarly oriented repository as ILDgenDB, utilizing genomic, transcriptomic, proteomic and drug information for interstitial lung diseases. ILDGDB incorporates 2018 entries for 20 ILDs and over 600 genes; its purpose is to decipher gene mechanisms that take place in ILDs.

There are also repositories, especially for genomic data, that can be exploited for ILD research. Gene Expression Omnibus (GEO) is a very popular, public repository which accommodates thousands of gene expression datasets. There is a dataset containing gene expression from lung tissue with pulmonary sarcoidosis as well as from healthy lung tissue (GSE16538). GEO also contains expression profiling of lung tissues from patients with idiopathic pulmonary fibrosis (GSE2052). Another similar repository is BioGPS, which features gene expression signatures from numerous diseases and conditions. In terms of ILDs, BioGPS contains two relevant gene sets: one for IPF (E-GEOD-24206) and another one for ILDs in general (E-GEOD-21369). The former was derived from 23 samples, while the latter was derived from 29; both contain data from healthy controls.

## 3. AI Applications in ILD Research

Applications of AI in ILD research can be roughly summarized in the following broad categories: (i) screening, (ii) diagnosis and classification, (iii) prognosis. The first category contains articles that aim to identify interstitial abnormalities before they become clinically meaningful and detectable. They focus primarily on interstitial lung abnormalities (ILAs) and exploit AI algorithms for their early identification, with the majority using CT scans as input. The second category, which is the most populated one, contains algorithms that diagnose ILDs mostly from imaging modalities, and some of them further aim to differentiate among various entities. The vast majority of studies in the last category (i.e., prognosis) deal with fibrosis and aim to assess its progression over time and subsequent prognosis. A summarizing table (Table 1) for the included studies has been added at the end of this section.

### 3.1. Screening

The most common approach in this category involves the analysis of imaging modalities for the identification of ILAs. Bermejo-Peláez et al. [11] analyze 208 CT scans, broken down to 37,424 radiographic tissue samples, and utilize an ensemble of deep convolutional neural networks. Their aim is to identify radiographic patterns that precede the development of ILDs and classify them into eight different parenchymal feature classes, namely: normal parenchyma, five interstitial patterns (ground-glass, reticular, nodular, linear scar, subpleural line) and two emphysematous patterns (centrilobular and paraseptal). The authors report very good performance with an average sensitivity of 91.41% and average specificity of 98.18%. Agarwala et al. [12] employ 168 CT scans, coming both from a public database and a private one, and use a convolutional neural network (CNN) to identify three radiographic patterns (i.e., consolidation, emphysema and fibrosis), yielding acceptable performance, Similarly, Kim et al. [13] aim to identify ILAs in routine CT scans, exhibiting 90.5% overall accuracy. Since CT scans, and especially HRCTs (high-resolution CT scans) are not readily available in all facilities, Nishikiori et al. [14] propose a deep-learning-based algorithm which assigns scores to chest X-rays, representing the probability of fibrosing interstitial lung diseases. The performance of the algorithm was further validated with CT scans and its detection capability was not inferior to that of doctors (including pulmonologists and radiologists).

A methodologically different approach was proposed by Onishchenko et al. [15], who systematically searched electronic health records for comorbidity patterns that were potentially associated with the development of IPF. Interestingly, the proposed non-invasive methodology can predict IPF from 1 to 4 years prior to definite diagnosis, with Area under the ROC Curve (AUC) > 0.84. The proposed algorithm can be applied in large cohorts, even in primary care, enabling early diagnosis with potentially better outcomes.

Another interesting approach was presented by Axelsson et al. [16], who aimed to identify proteins in circulating blood that are associated with ILAs. More than 4700 protein analytes from two major databases with more than 10,000 patients combined were searched for potential association with the incidence and progression of ILAs. The extracted protein markers were subsequently coupled with machine learning algorithms in order to identify interstitial lung disease early and predict its progression.

### 3.2. Diagnosis and Classification

Some of the first articles to engage AI in ILD research comprise this category, which is also the most populated one. The majority utilize machine learning algorithms, and primarily deep-learning-based ones, in order to analyze CT scans and identify regions pertaining to interstitial lung diseases [17,18]. Pawar and Talbar [17] propose a two-stage approach: the first is for segmenting HRCT images and the second is for classifying segmented images into six ILD classes (normal, emphysema, fibrosis, ground glass, micronodules and consolidation). The proposed classifier yielded an overall accuracy of 89.39%. Huang et al. [18] employ a dataset consisting of 108 annotated HRCT images and classified regions in five ILD-relevant classes (healthy, ground glass, emphysema, micronodules and fibrosis) with an F1-score of more than 0.96.

Besides region classification, some studies go further to provide a specific diagnosis [19,20]. Chloe et al. [19] developed a content-based image retrieval algorithm in order to aid the diagnosis of ILDs. The algorithm was evaluated on images from approximately 300 patients belonging to four categories and yielded overall accuracy of 60.9%. The four categories were: usual interstitial pneumonia (UIP), nonspecific interstitial pneumonia (NSIP), cryptogenic organizing pneumonia, and chronic hypersensitivity pneumonitis. In a methodologically different approach but for the same purpose Koo et al. [20] achieved a slightly better performance.

Among ILDs, IPF attracts considerable interest since it is relatively frequent and also bears quite unfavorable prognosis. For this purpose various approaches have been presented in the literature [21,22,23]. In a similar manner with the previous approaches, these articles describe AI based approaches for segmenting and classifying images, with special focus on IPF related patterns. All three studies utilized deep learning algorithms and were developed with 1068, 105 and 1239 patients, reporting overall accuracy 83.6%, 81%, while the latter one [23] reported an AUC = 0.87. Yang et al. [24] focused on another popular ILD entity, i.e., pneumoconiosis, and analyzed 1760 chest X-rays with a deep learning algorithm in order to discriminate between patients with pneumoconiosis and normal ones. The best performing algorithm resulted in 92.46% accuracy, which is significantly higher compared to other machine learning algorithms (e.g., support vector machines, artificial neural networks, random forests, k-nearest neighbors) used in the same study.

A totally different approach was presented by Horimasu et al. [25], who developed a machine learning algorithm in order to discriminate fine crackles from other auscultatory sounds and subsequently associate them with respective regions of pulmonary fibrosis. In total, 60 patients underwent chest X-ray and HRCT, and respiratory findings were recorded in six positions. Fine crackles were mostly associated with honeycombing and traction bronchiectasis; another interesting finding was that the identification of fine crackles indicated a higher sensitivity compared to a chest-X-ray-based determination of the presence or absence of ILDs.

In a recently published pilot study, Plantier et al. [26] analyzed the exhaled air from three patient categories, namely, patients diagnosed with IPF, patients with ILD secondary to connective tissue diseases (CTDs) and healthy controls. The aim of the study was to identify volatile organic compound patterns in exhaled air to non-invasively discriminate IPF- and CTD-related ILDs. The study employed approximately 150 patients and resulted in classification accuracy of 77.5%, sensitivity of 76.5% and specificity of 78.4%.

Zhang et al. [27] utilized a machine learning algorithm aiming to identify biomarkers from gene expression profiles that differentiate chronic hypersensitivity pneumonitis (CHP) from other ILDs. Based on the analysis of approximately 300 patients, the authors reported 674 CHP biomarkers with an ultimate aim to facilitate precise gene therapy for CHP. In a similar approach that was recently published, Li et al. [28] identified a six-gene subset whose expression was significantly different in patients with IPF compared to healthy controls. A random forest algorithm was used upon a set of more than 600 patients, yielding an AUC of 0.856.

### 3.3. Prognosis

From another perspective, there is the use of AI and deep learning for the prognosis of the disease, either for the progression of ILD or the possibility of malignancy development or coexistence. Different tools were created mostly based on quantitative imaging from HRCT, and some quantitative interstitial scores have also been developed. Kim et al. [29] propose that the use of imaging features of quantitative lung fibrosis scores in patients with IPF in the early stages can predict the progressive fibrosis status later on. The ability to apply this in precisely informed and timely management decisions after treatment is progress. AI could be used in testing new safe and effective therapies and to elucidate the effects of therapies in patients with biologically heterogeneous disease in the timing of progression. New software such as the artificial-intelligence-based quantitative CT image analysis software (AIQCT) of Handa et al. quantified parenchymal lesions and airway volumes; they suggest that lung volume on HRCT imaging of the chest may provide additional prognostic information on the gender–age–lung physiology stage of IPF [30].

Since patients with idiopathic pulmonary fibrosis are at a higher risk of developing lung cancer, radiomics could be an early prognostic indicator. Budzikowski et al. correlate image features with patients’ genetic mutations [31]. It is stated that these imaging features may serve as prognostic indicators combining radiomic features and genetic mutations. That also provides an understanding of the interaction between imaging phenotype and patient genotype on the progression and, furthermore, the treatment of IPF. Additionally, regarding the risk of malignancy development or coexistence, in a recent study of Liang et al., it is mentioned that whole-lung CT texture analysis is a promising tool for the lung cancer risk stratification of IPF patients [32]. Moreover, Aoki et al. [33] using deep-learning-based analysis and measured consolidation with fibrosis, found that it was independently associated with poor survival. They also found that the lesion extent measured using deep-learning-based analysis showed a negative correlation with pulmonary function test results and prognosis.

Another domain that AI is applied to is the investigation of the proteomic signature of ILD. The exact correspondence of cell state changes in diseased organs to peripheral protein signatures currently remains unknown. Certain plasma biomarkers in patients with progressive fibrosing ILD have been identified, and consistent associations across ILD subtypes have been reported. In a recent research study [34], a proteomic signature comprising 12 biomarkers was derived via machine learning, suggesting that approximately 10% of patients with a low-risk proteomic signature would experience ILD progression in the year after blood draw. Finally, Mayr et al. [35], using cross-modal analysis and machine learning, identified the cellular source of biomarkers. They demonstrated that information transfer between modalities predicts disease status. So, they also suggest the feasibility of clinical cell state monitoring through the longitudinal sampling of body fluid proteomes.

## 4. Discussion

AI algorithms are data driven; therefore, the reported outcomes are largely dependent on the quality and quantity of the input data. In this sense, if the data used to train the algorithms are biased, the outcomes will be biased as well. For example, movie recommendation services assess one’s taste and propose mostly similar movies, thus ignoring a great deal of genres and essentially leading to bias. Projecting this kind of bias onto search engine results or newsfeeds points to further ethical issues. In the case of ILD research, where the entities are relatively rare, it is important to obtain large amounts of high-quality data, where all entities are represented adequately, in order to avoid such bias.

Another significant challenge in the utilization of AI algorithms in healthcare is the difficulty of capturing and representing contextual information. For example, the process of taking medical history is difficult to model in an automated manner; this issue becomes even more evident in complex cases where different aspects of the condition emerge progressively as more exams are performed and results become available.

Imaging modalities and especially high-resolution CT scans coupled with lung function testing constitute the pillars for ILD diagnosis and progression monitoring, and the respiratory physician (or ILD specialist) must be well trained in both. Radiomics is an interesting and forthcoming field in interstitial lung diseases. Within the field of medical imaging, radiomics refers to the methodologies for extracting minable data from medical images, which is crucial in the field of the current review article. Despite advances in imaging modalities, the extracted features are susceptible to variation across scanner hardware and acquisition protocols. For this purpose, there is active discussion in the literature in terms of harmonization solutions aimed at minimizing this variation; these solutions are roughly divided into two categories: image domain and feature domain [36]. The former category includes methods applied on the whole image prior to feature extraction (e.g., standardization of image acquisition, data augmentation, etc.). The latter category (i.e., feature domain), utilizes methods applied during or after feature extraction, aiming to harmonize the extracted radiomic features. In the overall data harmonization process, the impact of deep learning should also be highlighted, since its algorithms are able to ingest large amounts of raw unstructured data and essentially automate the feature extraction process [37].

Many studies employ different imaging modalities in ILDs, mostly HRCT, but some employ chest X-rays, and others, the combination of both. The utilization of chest X-rays either alone or in a complementary manner with other sources of data (e.g., auscultation) denotes a very interesting prospect, since CT scans are not readily available in all healthcare facilities. On the other hand, chest X-rays are available in almost all facilities, very quickly with minimal cost and negligible radiation. Radiomics can also be combined with other biomarkers such as plasma proteins and can be used to identify early lung malignancy among ILD patients. Additionally, the use of VOCs or gene expression profiles needs to be further investigated and standardized due to the complexity of radiomics.

From an algorithmic point of view, primarily deep learning algorithms are employed, especially in most recent articles. Since the majority of studies in the field of ILDs utilize imaging data, deep learning algorithms are a very appealing choice. Deep learning algorithms have been extensively used in several fields with very promising results; especially in the case of image analysis, they have proven particularly effective with minor interference from field experts. A long-standing issue affecting the acceptance of AI algorithms in healthcare is the explainability of the reported results. For this purpose in earlier years, primarily decision-tree-based algorithms were employed, where the results were relatively easily comprehensible. Even though the complex architecture of deep learning algorithms makes explainability quite elusive, there have been tremendous efforts to provide some reasoning for the reported outcomes. Nevertheless, the impressive performance of deep learning algorithms has contributed to their acceptance, despite lack of explainability.

Almost all articles mentioned in this review used deep learning algorithms, as we have focused on studies published within the last 3–4 years, i.e., from 2019–2023. In these, relatively few datasets are used, and many authors validate algorithms on private datasets. The results reported from the majority of the studies included in the current review article seem very promising; especially in studies utilizing imaging modalities, rigorous validation has been performed, oftentimes using large databases, e.g., the OSIC data repository and medGIFT. Nevertheless, translation of these radiological analyses into clinical practice progresses slowly due to several challenges pertaining to workflow standardization and reproducibility.

Within the current review article, there is a notable lack of AI approaches focusing on the treatment of ILDs. There are, however, plenty of articles assessing the severity and prognosis of ILDs, especially IPF, indirectly guiding the subsequent therapeutic plan. Recent advances in the AI field, coupled with an increasing understanding of ILD biomarkers, are expected to facilitate the shift towards precision medicine in ILDs, with personalized, AI-directed treatments [6,38]. Moreover, a novel drug target for IPF was recently discovered via a generative AI platform, yet this prospect remains to be further explored [39].

As more and more AI applications in healthcare and ILD research in particular become available with promising results, their deployment in clinical practice needs to be orchestrated. This issue calls for administrative and regulatory compliance. To this end, the US Food and Drug Administration (FDA) has been increasingly issuing approval for such AI tools during the last few years. Another important issue regarding utilization in clinical practice is to obtain reimbursement from insurance companies for AI tools.

During the COVID-19 pandemic, a boost in automatic analysis of CT scans was noted. It should be mentioned that COVID-19 has accelerated the utilization of both health informatics and AI. Due to the easy transmission of the virus, telehealth devices for remote monitoring were widely employed. Moreover, the characteristic patterns depicted in CT scans yielded several AI algorithms for the detection and quantification of lung damage in COVID patients. The overwhelming difficulty of evaluating patients in person, especially for respiratory conditions, has catalyzed the development of novel means to evaluate, diagnose and monitor such patients. Among others, fibrosis following COVID infection was a new topic of research, but also, many incidental findings such as ILAs were discovered with this extensive use of CT for COVID patients. There are many recent studies about the management of and research on interstitial lung abnormalities and their role in ILDs. These findings are included in new datasets processed using AI in order to maximize the significance of the results. Due to many entities, almost all studies focus on radiologic patterns (emphysema, etc.), and if they delve into diagnosis, most of them involve IPF and scarcely mention chronic hypersensitivity pneumonitis. To the best of our knowledge, intriguingly, there were no recent articles regarding cryptogenic organizing pneumonia (COP), which could be a field for further research. There is a group of articles analyzing ILDs related to autoimmune diseases that are not included in this review.

As mentioned earlier, due to the nature of ILDs, there are relatively few ILD specialists, often leading to considerable delay in reaching an expert and thus obtaining a definitive diagnosis. There is a need to create helpful tools and provide knowledge through AI and advanced AI decision support systems worldwide. During the COVID era, large databases were developed especially for imaging, and there has been significant progress in AI pertaining to radiology. Respiratory medicine follows the broad use of AI in medicine, depicting the need for accurate, correct and quick solutions.

### Future Directions

The use of AI and especially deep learning in the analysis of imaging modalities in interstitial lung diseases has been on the rise during the last few years and is far from being saturated. Imaging biomarkers are being identified and correlated with disease outcomes, but also, advanced AI techniques are being utilized with increased generalization capability. Another important factor affecting the acceptance of AI techniques in ILD research, and medicine in general, is the explainability of the reported outcomes, which is gradually becoming available for many algorithms. Nevertheless, the complex nature of ILDs’ pathobiology, which encompasses both environmental and genetic interactions, makes the utilization of AI algorithms ideal for this elusive purpose. Another interesting prospect for the use of AI in ILD research is the exploration and characterization of ILAs, which are recently in the spotlight. Overall, same as in several medical fields, the implementation of AI techniques in clinical practice is underway, aiming for maximal patient benefit.

## Figures and Tables

**Table 1:** Summary of the studies included in the current review article.

Author(s)	Scope	Dataset	Type of Data	Performance
Bermejo-Peláez et al. [11]	Screening	208 CT scans	Imaging	Sensitivity: 91.41%
Agarwala et al. [12]	Screening	168 CT scans	Imaging	Success rate: 85.3%
Kim et al. [13]	Screening	336 participants	Imaging	Accuracy: 90.5%
Nishikiori et al. [14]	Screening	1159 chest X-rays	Imaging	AUC = 0.979
Onishchenko et al. [15]	Screening	2,983,215 participants	Electronic Health Records	AUC > 0.840
Axelsson et al. [16]	Screening	>10,000 patients	Proteins	-
Pawar and Talbar [17]	Diagnosis & classification	108 CT scans	Imaging	Accuracy: 89.39%
Huang et al. [18]	Diagnosis & classification	108 CT scans	Imaging	F1-score > 0.96
Chloe et al. [19]	Diagnosis & classification	300 patients	Imaging	Accuracy: 60.9%
Koo et al. [20]	Diagnosis & classification	1085 patients	Imaging	AUC > 0.900
Furukawa et al. [21]	Diagnosis & classification	1068 patients	Imaging	Accuracy: 83.6%
Christe et al. [22]	Diagnosis & classification	105 patients	Imaging	Accuracy: 81%
Bratt et al. [23]	Diagnosis & classification	1239 patients	Imaging	AUC = 0.870
Yang et al. [24]	Diagnosis & classification	1760 chest X-rays	Imaging	Accuracy: 92.46%
Horimasu et al. [25]	Diagnosis & classification	60 patients	Auscultation	Accuracy: 75%
Plantier et al. [26]	Diagnosis & classification	150 patients	Volatile Organic Compounds	Accuracy: 77.5%
Zhang et al. [27]	Diagnosis & classification	300 patients	Gene Expression	-
Li et al. [28]	Diagnosis & classification	600 patients	Gene Expression	AUC = 0.856
Kim et al. [29]	Prognosis	192 patients	Imaging	-
Handa et al. [30]	Prognosis	465 patients	Imaging	-
Budzikowski et al. [31]	Prognosis	169 patients	Imaging and Genomic	-
Liang et al. [32]	Prognosis	116 patients	Imaging	AUC = 0.870
Aoki et al. [33]	Prognosis	104 patients	Imaging	-
Bowman et al. [34]	Prognosis	589 patients	Proteomic	Sensitivity: 90%
Mayr et al. [35]	Prognosis	124 patients	Proteomic	Accuracy: 83%

## Data Availability

Data sharing not applicable.

## References

[1] Wijsenbeek M., Suzuki A., Maher T.M. (2022). Interstitial Lung Diseases. Lancet.

[2] Cosgrove G.P., Bianchi P., Danese S., Lederer D.J. (2018). Barriers to Timely Diagnosis of Interstitial Lung Disease in the Real World: The INTENSITY Survey. BMC Pulm. Med..

[3] Gouda W., Sama N.U., Al-Waakid G., Humayun M., Jhanjhi N.Z. (2022). Detection of Skin Cancer Based on Skin Lesion Images Using Deep Learning. Healthcare.

[4] Luo X., Zhang J., Li Z., Yang R. (2022). Diagnosis of Ulcerative Colitis from Endoscopic Images Based on Deep Learning. Biomed. Signal Process. Control.

[5] Shamai G., Livne A., Polónia A., Sabo E., Cretu A., Bar-Sela G., Kimmel R. (2022). Deep Learning-Based Image Analysis Predicts PD-L1 Status from H&E-Stained Histopathology Images in Breast Cancer. Nat. Commun..

[6] Maher T.M., Nambiar A.M., Wells A.U. (2022). The Role of Precision Medicine in Interstitial Lung Disease. Eur. Respir. J..

[7] Mandal S., Balas V.E., Shaw R.N., Ghosh A. Prediction Analysis of Idiopathic Pulmonary Fibrosis Progression from OSIC Dataset. Proceedings of the 2020 IEEE International Conference on Computing, Power and Communication Technologies (GUCON).

[8] Depeursinge A., Vargas A., Platon A., Geissbuhler A., Poletti P.-A., Müller H. (2012). Building a Reference Multimedia Database for Interstitial Lung Diseases. Comput. Med. Imaging Graph..

[9] Mishra S., Shah M.I., Sarkar M., Asati N., Rout C. (2018). ILDgenDB: Integrated Genetic Knowledge Resource for Interstitial Lung Diseases (ILDs). Database.

[10] Li Y., Wu G., Shang Y., Qi Y., Wang X., Ning S., Chen H. (2020). ILDGDB: A Manually Curated Database of Genomics, Transcriptomics, Proteomics and Drug Information for Interstitial Lung Diseases. BMC Pulm. Med..

[11] Bermejo-Peláez D., Ash S.Y., Washko G.R., San José Estépar R., Ledesma-Carbayo M.J. (2020). Classification of Interstitial Lung Abnormality Patterns with an Ensemble of Deep Convolutional Neural Networks. Sci. Rep..

[12] Agarwala S., Kale M., Kumar D., Swaroop R., Kumar A., Kumar Dhara A., Basu Thakur S., Sadhu A., Nandi D. (2020). Deep Learning for Screening of Interstitial Lung Disease Patterns in High-Resolution CT Images. Clin. Radiol..

[13] Kim M.S., Choe J., Hwang H.J., Lee S.M., Yun J., Kim N., Ko M.-S., Yi J., Yu D., Seo J.B. (2022). Interstitial Lung Abnormalities (ILA) on Routine Chest CT: Comparison of Radiologists’ Visual Evaluation and Automated Quantification. Eur. J. Radiol..

[14] Nishikiori H., Kuronuma K., Hirota K., Yama N., Suzuki T., Onodera M., Onodera K., Ikeda K., Mori Y., Asai Y. (2023). Deep-Learning Algorithm to Detect Fibrosing Interstitial Lung Disease on Chest Radiographs. Eur. Respir. J..

[15] Onishchenko D., Marlowe R.J., Ngufor C.G., Faust L.J., Limper A.H., Hunninghake G.M., Martinez F.J., Chattopadhyay I. (2022). Screening for Idiopathic Pulmonary Fibrosis Using Comorbidity Signatures in Electronic Health Records. Nat. Med..

[16] Axelsson G.T., Gudmundsson G., Pratte K.A., Aspelund T., Putman R.K., Sanders J.L., Gudmundsson E.F., Hatabu H., Gudmundsdottir V., Gudjonsson A. (2022). The Proteomic Profile of Interstitial Lung Abnormalities. Am. J. Respir. Crit. Care Med..

[17] Pawar S.P., Talbar S.N. (2022). Two-Stage Hybrid Approach of Deep Learning Networks for Interstitial Lung Disease Classification. BioMed Res. Int..

[18] Huang S., Lee F., Miao R., Si Q., Lu C., Chen Q. (2020). A Deep Convolutional Neural Network Architecture for Interstitial Lung Disease Pattern Classification. Med. Biol. Eng. Comput..

[19] Choe J., Hwang H.J., Seo J.B., Lee S.M., Yun J., Kim M.-J., Jeong J., Lee Y., Jin K., Park R. (2022). Content-Based Image Retrieval by Using Deep Learning for Interstitial Lung Disease Diagnosis with Chest CT. Radiology.

[20] Koo C.W., Williams J.M., Liu G., Panda A., Patel P.P., Frota Lima L.M.M., Karwoski R.A., Moua T., Larson N.B., Bratt A. (2022). Quantitative CT and Machine Learning Classification of Fibrotic Interstitial Lung Diseases. Eur. Radiol..

[21] Furukawa T., Oyama S., Yokota H., Kondoh Y., Kataoka K., Johkoh T., Fukuoka J., Hashimoto N., Sakamoto K., Shiratori Y. (2022). A Comprehensible Machine Learning Tool to Differentially Diagnose Idiopathic Pulmonary Fibrosis from Other Chronic Interstitial Lung Diseases. Respirology.

[22] Christe A., Peters A.A., Drakopoulos D., Heverhagen J.T., Geiser T., Stathopoulou T., Christodoulidis S., Anthimopoulos M., Mougiakakou S.G., Ebner L. (2019). Computer-Aided Diagnosis of Pulmonary Fibrosis Using Deep Learning and CT Images. Investig. Radiol..

[23] Bratt A., Williams J.M., Liu G., Panda A., Patel P.P., Walkoff L., Sykes A.-M.G., Tandon Y.K., Francois C.J., Blezek D.J. (2022). Predicting Usual Interstitial Pneumonia Histopathology from Chest CT Imaging With Deep Learning. Chest.

[24] Yang F., Tang Z.-R., Chen J., Tang M., Wang S., Qi W., Yao C., Yu Y., Guo Y., Yu Z. (2021). Pneumoconiosis Computer Aided Diagnosis System Based on X-Rays and Deep Learning. BMC Med. Imaging.

[25] Horimasu Y., Ohshimo S., Yamaguchi K., Sakamoto S., Masuda T., Nakashima T., Miyamoto S., Iwamoto H., Fujitaka K., Hamada H. (2021). A Machine-Learning Based Approach to Quantify Fine Crackles in the Diagnosis of Interstitial Pneumonia: A Proof-of-Concept Study. Medicine.

[26] Plantier L., Smolinska A., Fijten R., Flamant M., Dallinga J., Mercadier J.J., Pachen D., d’Ortho M.P., van Schooten F.J., Crestani B. (2022). The Use of Exhaled Air Analysis in Discriminating Interstitial Lung Diseases: A Pilot Study. Respir. Res..

[27] Zhang H., Wang S., Huang T. (2021). Identification of Chronic Hypersensitivity Pneumonitis Biomarkers with Machine Learning and Differential Co-Expression Analysis. Curr. Gene Ther..

[28] Li Z., Wang S., Zhao H., Yan P., Yuan H., Zhao M., Wan R., Yu G., Wang L. (2023). Artificial Neural Network Identified the Significant Genes to Distinguish Idiopathic Pulmonary Fibrosis. Sci. Rep..

[29] Kim G.H.J., Weigt S.S., Belperio J.A., Brown M.S., Shi Y., Lai J.H., Goldin J.G. (2020). Prediction of Idiopathic Pulmonary Fibrosis Progression Using Early Quantitative Changes on CT Imaging for a Short Term of Clinical 18-24-Month Follow-Ups. Eur. Radiol..

[30] Handa T., Tanizawa K., Oguma T., Uozumi R., Watanabe K., Tanabe N., Niwamoto T., Shima H., Mori R., Nobashi T.W. (2022). Novel Artificial Intelligence-Based Technology for Chest Computed Tomography Analysis of Idiopathic Pulmonary Fibrosis. Ann. Am. Thorac. Soc..

[31] Budzikowski J.D., Foy J.J., Rashid A.A., Chung J.H., Noth I., Armato S.G. (2021). 3rd Radiomics-Based Assessment of Idiopathic Pulmonary Fibrosis Is Associated with Genetic Mutations and Patient Survival. J. Med. Imaging Bellingham.

[32] Liang C.-H., Liu Y.-C., Wan Y.-L., Yun C.-H., Wu W.-J., López-González R., Huang W.-M. (2021). Quantification of Cancer-Developing Idiopathic Pulmonary Fibrosis Using Whole-Lung Texture Analysis of HRCT Images. Cancers.

[33] Aoki R., Iwasawa T., Saka T., Yamashiro T., Utsunomiya D., Misumi T., Baba T., Ogura T. (2022). Effects of Automatic Deep-Learning-Based Lung Analysis on Quantification of Interstitial Lung Disease: Correlation with Pulmonary Function Test Results and Prognosis. Diagnostics.

[34] Bowman W.S., Newton C.A., Linderholm A.L., Neely M.L., Pugashetti J.V., Kaul B., Vo V., Echt G.A., Leon W., Shah R.J. (2022). Proteomic Biomarkers of Progressive Fibrosing Interstitial Lung Disease: A Multicentre Cohort Analysis. Lancet Respir. Med..

[35] Mayr C.H., Simon L.M., Leuschner G., Ansari M., Schniering J., Geyer P.E., Angelidis I., Strunz M., Singh P., Kneidinger N. (2021). Integrative Analysis of Cell State Changes in Lung Fibrosis with Peripheral Protein Biomarkers. EMBO Mol. Med..

[36] Mali S.A., Ibrahim A., Woodruff H.C., Andrearczyk V., Müller H., Primakov S., Salahuddin Z., Chatterjee A., Lambin P. (2021). Making Radiomics More Reproducible across Scanner and Imaging Protocol Variations: A Review of Harmonization Methods. J. Pers. Med..

[37] Hinton G. (2018). Deep Learning—A Technology with the Potential to Transform Health Care. JAMA.

[38] Antoniou K.M., Tsitoura E., Vasarmidi E., Symvoulakis E.K., Aidinis V., Tzilas V., Tzouvelekis A., Bouros D. (2021). Precision Medicine in Idiopathic Pulmonary Fibrosis Therapy: From Translational Research to Patient-Centered Care. Curr. Opin. Pharmacol..

[39] Mak K.-K., Balijepalli M.K., Pichika M.R. (2022). Success Stories of AI in Drug Discovery—Where Do Things Stand?. Expert Opin. Drug Discov..

